# Caregivers Use Joint Attention to Support Sign Language Acquisition in Deaf Children

**DOI:** 10.1111/desc.70034

**Published:** 2025-05-29

**Authors:** Jennifer Sander, Caroline F. Rowland, Amy M. Lieberman

**Affiliations:** ^1^ Max Planck Institute for Psycholinguistics Nijmegen the Netherlands; ^2^ Max Planck School of Cognition Leipzig Germany; ^3^ Donders Institute for Brain, Cognition and Behaviour Radboud University Nijmegen the Netherlands; ^4^ Wheelock College of Education & Human Development Boston University Boston Massachusetts USA

**Keywords:** ASL, dyadic interaction, joint attention, language development, sign language, visual attention

## Abstract

Children's ability to share attention with another social partner (joint attention) plays an important role in language development. However, our understanding of the role of joint attention comes mainly from children learning spoken languages, which gives a very narrow, speech‐centric impression of the role of joint attention. This study broadens the scope by examining how deaf children learning a sign language achieve joint attention with their caregivers during natural social interaction, and how caregivers provide word learning opportunities. We analyzed naturalistic play sessions of 54 caregiver‐child dyads using American Sign Language (ASL), and identified joint attention that surrounded caregivers’ labeling of either familiar or novel objects using a comprehensive multimodal coding scheme. We observed that dyads using ASL establish joint attention using linguistic, visual, and tactile cues, and that most naming events took place in the context of a successful joint attention episode. Key characteristics of these joint attention episodes were significantly correlated with the children's expressive vocabulary size, mirroring the patterns observed for spoken language acquisition. We also found that sign familiarity as well as the order of mention of object labels affected the timing of naming events within joint attention. Our results suggest that caregivers using ASL are highly sensitive to their child's visual attention in interactions and modulate joint attention differently when providing familiar versus novel object labels. These joint attentional episodes facilitate word learning in sign language, just as they do in spoken language interactions.

## 1 Introduction

Joint attention (JA), described as the active, shared coordinated attention of two people on an object or an event of interest (Gabouer and Bortfeld 2021), plays a critical role in the development of language, cognition, and social skills in children (Carpenter et al. 1998; Tomasello 2014; Mundy and Newell 2007). In particular, there is a growing consensus that joint attentional interactions play a key role in the early stages of language acquisition (see Çetinçelik et al. 2021). However, our current understanding of joint attention and its role in language learning is based largely on interactions that occur via spoken language, in which there is simultaneous input of auditory linguistic (spoken object labels) and visual non‐linguistic (visual attention on the object) information (e.g., Tomasello and Farrar 1986). This means that most theories implicitly or explicitly assume that joint attention facilitates language learning because joint attention episodes occur during dyadic interactions where both partners are looking at the referent object at the time of naming such that there is simultaneity of inputs.

**Summary**

- Joint attention between deaf children using American Sign Language (ASL) and their caregivers is achieved through multimodal behaviors including gaze, touch, and sign.
- Caregivers interacting with deaf children are highly sensitive to children's gaze when labeling objects.
- Caregivers adjust the timing of object labels based on whether they are labeling a familiar or novel object.
- Children's engagement in joint attention in ASL correlates with their expressive ASL vocabulary.

In this study, we investigated whether this definition of joint attention is too narrow—in other words, whether the simultaneity of visual attention to an object and language input is critical or whether other forms of JA including multimodal behaviors and non‐simultaneity can be equally facilitative for language acquisition. We do so by exploring how deaf children learning a sign language achieve joint attention with their caregivers during natural sign interactions, and by investigating whether, and how, these joint attentional episodes scaffold word learning opportunities for children.

During a canonical joint attentional episode in a spoken language interaction, the child and the adult are both attending to a referent (e.g., an object or event) at the precise moment at which the adult provides the label used to describe that referent. This makes the mapping between the label and the object highly salient, resulting in what some have argued is an ideal word learning moment (e.g., Whitehurst et al. 1982; Tomasello and Farrar 1986). Several studies support this thesis that these joint attention episodes provide such word learning moments, both by demonstrating that children who engage in more and longer joint attentional episodes tend to have faster vocabulary growth (Carpenter et al. 1998; Morales et al. 1998; Brooks and Meltzhoff 2005; Yu et al. 2019) and by demonstrating more effective novel word learning during joint attention episodes in experimental settings (Moore et al. 1999; Graham et al. 2010; Hirotani et al. 2009).

However, this is a narrow definition of joint attention. It ignores the fact that there are many types of joint attentional interactions, and that these have different characteristics, take place in different cultural and social contexts, and/or in different populations, and may also provide key learning opportunities (see Akhtar and Gernsbacher 2007 for an illustration of the variety of joint attention realities). For example, word learning has been noted to occur outside canonical joint attentional contexts in interactions with autistic children (e.g., Mundy et al. 1990), children with Williams Syndrome (e.g., Laing et al. 2002), and typically developing populations (e.g., Akhtar et al. 2001). In addition, cultural differences in attentional patterns have been observed; for example, in Guatemalan Mayan toddlers (Correa‐Chávez and Rogoff 2009) compared to toddlers from Western Europe and North America, as well as cultural differences in the amount and type of direct input to children (Casillas et al. 2020, 2021).

In dyads interacting in a sign language such as American Sign Language (ASL), information about both linguistic and non‐linguistic information is perceived visually. Thus, signing children must learn to allocate visual attention sequentially in order to connect object labels to objects (e.g., to first look at the object and then at the sign, or vice versa; see Lieberman et al. 2022). Caregivers who are interacting with children using sign language must be sensitive to the child's visual attention in order to ensure that their input is perceived by the child (Gappmayr and Lieberman 2024; Pizer et al. 2008; Koester and McCray 2011), and to ensure that the child is able to connect the input to the corresponding referent (Lieberman et al. 2022; Leary et al. 2022). Caregivers can achieve this by using overt attention‐getting behaviors (such as tapping or waving), or waiting for the child to naturally shift gaze to the caregiver before signing (Swisher 1992; MacGowan et al. 2021). Signing dyads have also been observed to engage significantly more often in mutual gaze and show higher rates of gaze switches than dyads interacting in spoken language (Gale and Schick 2008; Lieberman et al. 2014).

While we know that the structure of gaze behaviors in signed interactions differs from that in spoken interactions, we do not know yet whether and how these changes in gaze behaviors affect the role of joint attention in early language development. However, since language acquisition proceeds on a typical timescale among deaf children who are exposed to a sign language from an early age (Newport and Meier 2017), we would expect that joint attention will be successfully achieved among signing dyads and that it will support word learning.

The goal of the present study was to investigate how deaf children learning ASL achieve joint attention with their caregivers during natural signed interaction and whether, and how, these joint attentional episodes scaffold word learning opportunities for children. First, we assessed how caregivers structure joint attentional interactions to support object labelling (i.e., naming events) in signed interactions and whether these joint attention episodes predict vocabulary acquisition. Second, we investigated whether and how joint attention differs around novel and familiar ASL naming events. Finally, within the novel naming events, we asked whether joint attention differs when naming an object for the first time in an interaction versus subsequent occurrences.

To achieve these aims, we needed an operationalization of joint attention that was suitable for coding joint attention in interactions using sign language. We adopted the coding scheme of Gabouer and Bortfeld (2021), which recognizes a number of joint attention behaviors beyond joint gaze. Using this scheme, we identified three different measures of joint attention: the success of joint attention initiation attempts, the duration of successful joint attention episodes, and the rate of successful joint attention initiation by the child. With regard to our first aim, we expected to find similar relationships between joint attention episodes and naming events in signing dyads as has been reported for speaking dyads; for example, that a substantial proportion of naming events would occur during successful joint attention episodes, and that there would be a relationship between the rate of successful joint attention initiation and vocabulary development. Further, we predicted that longer joint attention episodes would offer more possibilities to connect a label to the corresponding object, and, based on previous findings, that child‐initiated joint attention episodes would be most effective for sign learning (Tomasello and Todd 1983).

Our second aim was to ask whether caregivers systematically varied their approach to joint attention based on whether the object label was familiar or novel to the child. Joint attention has repeatedly been shown to facilitate novel word learning in spoken languages in experimental settings (see e.g., Hirotani et al. 2009). Further, it has been suggested that dyads using a spoken language modify their joint attentional behaviors depending on a label's novelty (Chen et al. 2021). It is unclear whether such a modification would also be observed for signing dyads. We were particularly interested here in how joint attention episodes surrounding naming events were initiated, because the initiation is the first element of a joint attention episode and constitutes the crucial bid for attention. According to the Gabouer and Bortfeld coding scheme, joint attention can be initiated using a range of visual and/or tactile behaviors including tapping the interaction partner, waving, using objects, or shifting gaze, or even the naming event itself (in this case an ASL sign), under the assumption that the sign itself will attract or maintain the child's attention. We hypothesized that caregivers would be particularly sensitive to the child's locus of attention, and would structure joint attention episodes differently when introducing novel and familiar object labels. In particular we expected caregivers to be sensitive to the high temporal demands of joint attention episodes in the context of signed interactions. We hypothesized that caregivers would establish joint attention prior to naming an object in ASL, in particular in the context of novel signs, to ensure the child can connect the novel sign to the object. When labeling a new object, the child's gaze to the caregiver during labeling is critical to the child's ability to learn the novel label and map it to the referent. We also expected caregivers to repeat novel signs more often to increase the number of possible instances the child can utilize to map the label to the novel object.

Finally, we explored whether the *first* mention of a novel object occurred within joint attention episodes that had unique properties relative to subsequent joint attention episodes. We reasoned that the first mention is actually the only truly novel object label, in that subsequent mentions repeat a label to which the child has now been exposed. We hypothesized that some characteristics of joint attention might be unique to the first mention of a novel object, specifically that caregivers would take extra care to ensure that joint attention had been successfully established prior to producing a novel label for the first time.

## 2 Method

### 2.1 Participants

Participants were 54 parent‐deaf child dyads whose primary language was ASL. Children were between the age of 9 months and 69 months (*M* = 38.6, SD = 14.2); 38 identified as male and 16 as female. All children were either deaf or hard of hearing (as reported by parents) and attended early childhood programs that used ASL. Parents were deaf (*n* = 44) or hearing (*n* = 10) and all reported using ASL with their deaf child. The majority of the parents identified themselves and their child as White (44 White, two Asian, one African American / Black, three more than one race, four did not give an answer; six parents identified their child's ethnicity as Hispanic or Latino). Participants represent a subset of dyads from the ASL‐PLAY database, a corpus of parent–child interactions in ASL previously collected by the last author (OSF‐link: https://osf.io/3w8ka/). Some dyads participated in multiple sessions; the first recording of dyads with more than one session was selected for the current study.

### 2.2 Materials and Procedure

Children played with a parent in a naturalistic play situation in a room at the child's school, or at a local setting. Dyads participated in one of two types of play sessions: familiar or novel. In *familiar* play sessions (*n* = 23, *M age* = 35 m.o.), dyads were given a set of toys representing common objects for which children in this age range are likely to have already acquired ASL signs. Items included a farm set, a Lego train set, a wooden fruit set, and a wooden car‐carrier truck. In *novel* play sessions (*n* = 31, *M age* = 41 m.o.), dyads were given a set of familiar objects (a set of toy animals and a wooden fruit set) as well as four novel objects: two animal figures (ostrich, armadillo) and two toy fruits (kiwi, dragonfruit). These items were chosen because they are unlikely to be known by the child and because they do not have common lexical signs in ASL. Signs for these four objects were borrowed from other sign languages (identified at spreadthesign.com) and introduced to the parents in advance so that parents could use the novel sign when interacting with the object during the play sessions.

Parents were instructed to play with their child as they typically would. In the novel sessions, parents were also instructed to use the provided sign labels for the novel objects when they encountered them. Each play session lasted between 12 and 15 min and was recorded from three different angles.

At the time of recording, children's ASL vocabulary was assessed through an online parental questionnaire, the ASL‐CDI 2.0 (Caselli et al. 2020), which is an adaptation of the Mac‐Arthur‐Bates Communicative Development Inventory (MB‐CDI; see Marchman and Dale (2023) for information about CDI construction reliability and validity). The ASL‐CDI 2.0 contains a total of 533 vocabulary items; parents see a video of each sign (and can click to see the written English gloss), and then select whether the child does not know, understands, or understands and produces the sign. In addition, parents can select whether their family uses a different sign for an item, and can skip unknown items. It is suitable for children aged between 8 months and up to 60 months of age, although it can be used with older children who were delayed in initial exposure to ASL. Proportional production and comprehension scores were derived (a proportion of all items known/produced by the child divided by the total number of completed items on the checklist). As a large number of children were at or near ceiling on vocabulary comprehension, the production score was used in the current analysis. We obtained results for this measure for 36 of the children.

### 2.3 Data Coding

Data was annotated in the audio‐visual annotation program ELAN (Version 6.4; MPI for Psycholinguistics, The Language Archive 2022). ASL signs were glossed using SignBank (Hochgesang et al. 2020) by fluent deaf signers, who also coded all attention‐getters. Child and parent gaze and object touches surrounding each ASL sign were coded by hearing fluent signers. From these annotations, we identified “naming events” in each interaction, defined as utterances in which the parent provided the label of an object that was available in the visual scene. In the familiar condition, we identified all naming events that referred to a concrete object present in the play session. In the novel condition, we identified all naming events that referred to one of the four novel objects; naming events of other objects during these novel play sessions were not coded.

We then applied the Gabouer and Bortfeld (2021) coding scheme for JA to annotate JA episodes surrounding each previously identified naming event. This coding scheme represents a flexible modality‐independent approach to identifying JA episodes in dyadic interactions, and is thus more suitable than classic, vision‐focused schemes. However, note that we did not annotate JA episodes that did not contain a naming event. Thus, the properties and characteristics of JA episodes we report are not the characteristics of JA episodes in general, but the characteristics of those JA episodes in which parents named objects with a familiar or novel sign.

**FIGURE 1:**
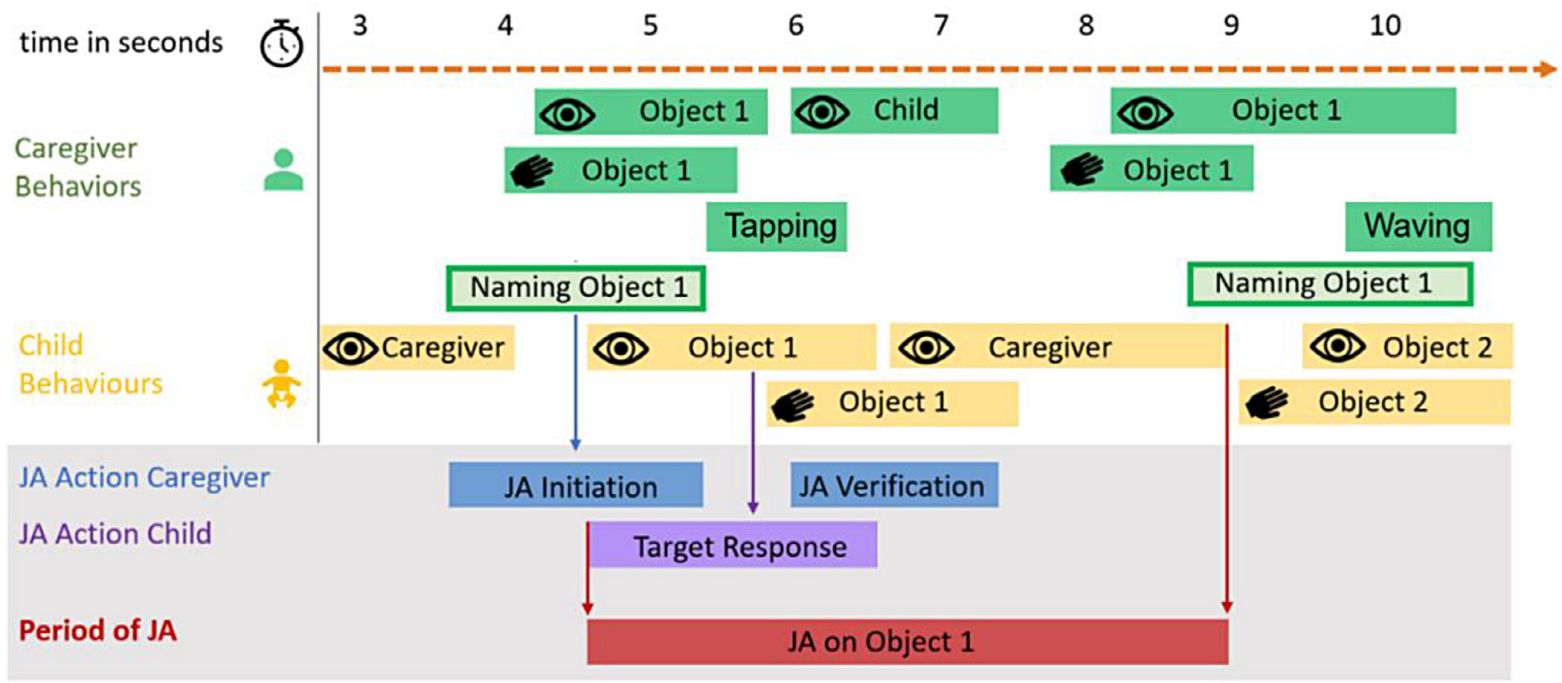
Schematic visualization of naturalistic data annotation from Gabouer and Bortfeld (2021). Caregiver (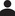, in green) and child behavior (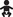, in yellow) is coded for gaze (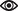), object touch (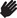), additional attention behaviors like waving or tapping, and naming events. Around Naming Events, joint attention is identified in a three‐step process. First, an initiation period is identified (blue), in this case the first object‐related behavior (naming the object) in which the initiator (in this case the caregiver) acted purposefully to share attention with the target (in this case the child). Second, the target response, if present, is identified (purple), in this case the first identified object‐related child behavior (gaze at the named object; (purple)). Third, the JA verification behavior (blue) is identified, in this case by the caregiver (looking at the child touching the object). The successfully initiated period of joint attention (red) has its onset with the onset of the target response and its offset with the offset of the last object or interaction partner‐related behavior (child gaze to caregiver) before a longer period of disengagement (5 s or longer). Time (in seconds) is visualized by the orange time stripe at the top. For successful joint attention to be present, all elements of the joint attention episode have to fulfill temporal requirements: the target response has to occur within 5 s after the offset of the joint attention initiation, and can involve several related behaviors but has to last in total at least 3 s. The joint attention verification then again has to take place at least 5 s after the onset of the target response. The time of shared attention also has to last at least 3 s for the joint attention episode to be considered successful.

Within the scheme, three behaviors must be present for an episode to be identified as successful JA between the two interaction partners: (1) an initiator's bid for attention; (2) a target's response to the bid; and (3) an initiator's verification of the target response. All three parts of the sequence must be present and in a specific temporal relationship to each other to be included: the target response has to take place within 5 s of the onset of the initiation and has to last in total at least 3 s, and the verification has to take place at least 5 s after the offset of the target response. The time of shared attention also has to last at least 3 s. If all of these criteria are met then the JA is considered successful. If any of these requirements are not fulfilled, the JA initiation attempt has not been successful in initiating JA and is considered an unsuccessful JA initiation attempt. Crucially, a number of different behaviors can be coded as initiation, target response, and verification, including touch, gaze, language usage, and attention‐getters like tapping and waving, as long as the behaviors are directed toward either the object of shared interest or toward the interaction partner and are produced with the intention to share attention. Behaviors can occur in isolation or combination (see Figure 1). Either interaction partner can take the role of the JA initiator or the role of the target of the JA initiation attempt. The JA is considered to begin at the onset of the target response and continues until the offset of the last object‐ or interaction partner‐related behavior before a longer period of disengagement (5 s or longer). Thus, while the initiation behavior is a critical criterion for establishing a joint attention episode, the coded JA episode does not include the initiation (see Figure 1).

We considered each relevant naming event, by definition, as at least a JA initiation bid, which resulted in two possible outcomes for each naming event: they could be either (1) part of a successful JA episode, or (2) part of an unsuccessful JA initiation bid.

We applied the coding scheme to identify all periods of JA episodes surrounding familiar and novel naming events. We extracted two categories of JA measures. The first category contained measures of JA that we anticipated would both predict vocabulary size and would differ between familiar and novel label conditions: (a) **JA initiation success rate** (proportion of initiations that led to successful JA), (b) **Mean JA duration** (the duration of successful JA in seconds), and (c) **Child JA initiation rate** (the proportion of successful JA episodes initiated by the child). We focused on the child for the third measure (c) because child‐led JA interactions have been shown to predict language development (e.g., Akhtar et al. 1991; McGillion et al. 2013), but note that these are simply the inverse of the proportion of caregiver‐initiated events.

The second category of measures identified the timing of the naming event within the JA episode, which we expected to differ between familiar and novel label conditions: (d) **Naming rate during JA initiation** (proportion of JA episodes in which naming occurred during the initiation step of JA); (e) **Time between JA onset and naming** (time between the onset of a JA episode and the first naming event in seconds; a negative value indicates the naming event started before the onset of JA (i.e., the naming event was part of the initiation bid)); and (f) **Naming frequency during JA** (the number of repetitions of the object label within each JA episode).

We defined sign novelty/familiarity by whether a familiar object was labelled in the familiar toy play sessions (**familiar condition**) or a novel item was labelled in the novel toy play sessions (**novel condition**). To determine whether the *first* mention of a novel object had unique properties relative to subsequent JA episodes, we identified for each dyad the first naming event of each object in either the familiar or novel session as a **first mention** and all subsequent naming events of the same objects as **subsequent mentions**.

## 3 Results

The data, as well as the code used for processing and analysis are available at: https://osf.io/wmeck/. All analyses were conducted in R‐Studio version 2023.12.0. (R Studio Team 2023). For all analyses, the significance level was set at 0.05 (two‐tailed), though, in the tables below, we also indicate if *p* < 0.01 and *p* < 0.001. The assumptions of all statistical tests were met without transformations unless otherwise stated.

### 3.1 How Dyads Structure Joint Attentional Episodes

Our first aim was to assess how dyads structure joint attentional interactions during naming events in signed interactions to support word learning. We identified a total of 882 naming events; of these, 720 naming events (81.6%) were embedded in a total of 521 successful JA episodes, as some JA episodes contained multiple naming events (repeated labelling of the same object). On average, there were 1.91 naming events per JA episode (SD = 1.2). There was high variability across dyads in the number of JA episodes (range 1 to 22 JA episodes), with an average of 6.98 JA episodes per dyad (SD = 5.07). There were small differences across the familiar and novel conditions: naming events were more likely to be embedded in JA episodes in the novel condition (87.05%; 336/386) than in the familiar condition (77.42%; 384/496).

In both familiar and novel conditions, most naming events were embedded in a successful JA episode and only in a small number of cases were they part of an unsuccessful JA initiation attempt (Table 1). A total of 144 JA initiation attempts did not successfully lead to a JA event. In the familiar condition, 31.34% (105/335) of the JA episode initiations were unsuccessful, compared to 20.97% (39/186) in the novel condition. Across conditions, 77 initiation *attempts* were unsuccessful; no period of shared attention was reached, as no target response to the JA initiation attempt was observed. For 63 JA initiations, a target response was observed and a corresponding period of shared attention was reached, but the episode did not reach the minimal requirement of three seconds of shared attention to be considered a JA episode. In four cases a period of shared attention was reached, but no verification behavior was observed.

**TABLE 1:** Number of naming events, number of Joint Attention (JA) initiations, duration of successful JA episodes, and proportion of JA episodes initiated by interaction partner by sign familiarity condition (familiar/novel).

Variable	Condition		
	Familiar condition	Novel condition	Total
Number of naming events			
Total	496	386	882
In successful JA episodes	384 (77.42%)	336 (87.05%)	720 (81.63%)
Number of JA initiations			
Total	335	186	521
Successful	230 (68.66%)	147 (79.03%)	377 (72.36%)
Mean duration of successful JA episodes (SD)	18.32 s (18.11 s)	17.37 s (16.7 s)	17.95 s (17.56 s)
Proportion of JA episode initiations by interaction partner			
Child	24.35%	14.29%	20.42%
Parent	75.65%	85.71%	79.58%

To initiate joint attention, show a target response or a verification, different behaviors could be employed. These were either visual (e.g., gaze following, signing, waving) or visual‐tactile (e.g., tapping, banging, object‐touch). A minority of these behaviors (e.g., banging, gasping) involved an auditory aspect, but their visual or tactile aspect would likely be more salient to the interlocuter. For joint attention initiation we observed signing and object manipulation to be the most common initiation behaviors, while for target response we observed gaze switches to be the most common behavior, and for verification gaze switches and signing were the most common behaviors.

### 3.2 JA and Vocabulary

Second, we assessed whether these joint attentional episodes predicted children's vocabulary acquisition. To do this we analyzed the relationship between the children's concurrent ASL vocabulary using ASL production score of the ASL‐CDI 2.0 and three separate measures of JA: JA initiation success, JA duration, and the proportion of JA initiated by the child. This analysis is based on the 36 children of our dataset for whom we obtained CDI scores.

**FIGURE 2:**
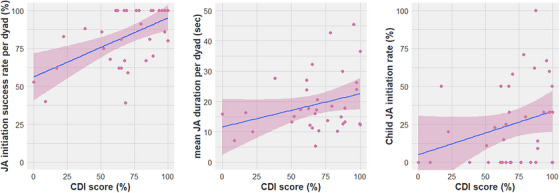
Relationship between production CDI scores and (a) JA initiation success rate per dyad, (b) mean JA duration, and (c) mean child JA initiation rate. Lines shows regression line, shading reflects 95% confidence interval, dots show scores for individual dyads.

We ran three regression models^1^ with expressive vocabulary size as the outcome measure, and, respectively, JA initiation success rate, mean JA duration, and child JA initiation rate as the predictor variables. Assuming that only successful JA episodes support language acquisition, the latter two models included successful JA episodes only. In all models, the assumptions of regression were met. The results are illustrated in Figure 2 and Table 2. There was a significant positive main effect of JA initiation success rate (*β* = 0.76, SE = 0.20, *t* = 3.78, *p* < 0.001) and of mean duration of successful JA episodes in seconds (*β* = 0.01, SE = 0.003, *t* = 2.32, *p* = 0.03), suggesting that children in dyads with higher average JA initiation success rates and with longer JA episodes had significantly higher concurrent ASL expressive vocabulary scores. Contrary to our predictions, there was no significant effect of child JA initiation rate on ASL vocabulary size (*β* = 0.27, SE = 0.16, *t* = 1.69, *p* = 0.10).

**TABLE 2:** Results of regression models: effect on production vocabulary (CDI) of (1) JA initiation success rate, (2) mean duration of successful JA episodes, and (3) Child JA initiation rate.

	Production vocabulary (CDI)					
Model	Predictors	Estimates	95% CI	SE	*t*	*p*
Model 1:	(Intercept)	0.06	[−0.29, 0.41]	0.17	0.35	0.73
	JA initiation success rate	0.76	[0.35, 1.17]	0.20	3.78	<0.001***
Model 2:	(Intercept)	0.56	[0.42, 0.71]	0.07	8.01	<0.001***
	Mean JA duration	0.01	[0.00, 0.01]	0.003	2.32	0.03*
Model 3:	(Intercept)	0.63	[0.51, 0.75]	0.06	10.74	<0.001***
	Child JA initiation rate	0.27	[‐0.05, 0.60]	0.16	1.69	0.10
Observations	36					
Model 1: AIC = −1.67, BIC = 3.08, *R* ^2^ = 0.3, RMSE = 0.22, *p* < 0.001***						
Model 2: AIC = 5.70, BIC = 10.45, *R^2^* = 0.14, RMSE = 0.24, *p* = 0.02*						
Model 3: AIC = 8.08, BIC = 12.83, *R* ^2^ = 0.08, RMSE = 0.25, *p* = 0.09						

*p < 0.05; **p < 0.01; ***p < 0.001.

### 3.3 JA in Novel and Familiar Object Naming Events

Our next aim was to determine whether caregivers systematically vary their approach to JA based on whether the object label is familiar or novel to the child. We compared the characteristics of JA episodes surrounding the two conditions: familiar (play sessions containing naming events of familiar objects) and novel (separate play sessions containing naming events of the four novel objects).

We identified a total of 521 JA episode initiations associated with the 720 naming events of our dataset, the majority of which (72.36%) culminated in a successful JA episode. In the familiar condition, 68.66% (230/335) of the JA episode initiations were successful, compared to 79% (147 out of 186) in the novel condition (Table 1). The average duration of a successful JA episode was 17.95 s (SD = 17.56 s) with JA episodes associated with familiar naming events being slightly longer (mean = 18.32 s, SD = 18.11 s) than those associated with novel naming events (mean = 17.37 s, SD = 16.7 s). Most successful JA episodes were initiated by the caregiver (mean = 80.31%, SD = 25.32%) with only a small number of JA episodes initiated by the child (mean = 19.69%, SD = 25.32%), and a higher proportion of JA episodes initiated by the child around familiar (24.35%) compared to novel (14.29%) naming events.

We ran three mixed effects models to analyze the effects of sign familiarity on our three JA outcome measures: **JA initiation success rate** (proportion of JA initiation bids leading to a JA episodes), **mean JA duration** (of successful JA in seconds), and **child JA Initiation rate** (proportion of successfully initiated JA episodes initiated by the child). Assuming that only successful JA episodes support language acquisition, the latter two models included successful JA episodes only.

**FIGURE 3:**
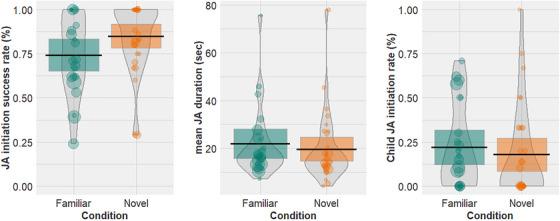
Relationship between sign familiarity (familiar vs. novel signs) and (a) JA initiation success rate per dyad (proportion of JA initiation bids leading to a JA episode), (b) mean JA duration per dyad (of successful JA in seconds), and (c) Child JA Initiation rate (proportion of successfully initiated JA episodes initiated by the child). Colors represent Condition (green = familiar, orange = novel). Points represent raw data from each dyad, point size represents number of JA episodes contributed per dyad. Bean shows smoothed density curve illustrating the full data distribution. Error bars show frequentist 95% confidence interval.

In all three models the predictor was the condition (familiar/novel), and we included random effects of dyad. For JA initiation success rate and child JA initiation rate we ran binomial generalized linear mixed effects models and for mean JA duration we ran a linear mixed effects model. Descriptive statistics for each condition are in Table 1 and Figure 3 and the results of the statistical models are illustrated in Table 3.

**TABLE 3:** Results for (generalized) linear mixed‐effects models: Effect of Condition on (1) JA initiation success rate (proportion of JA initiation bids leading to a JA episode), (2) mean JA duration per dyad (of successful JA in seconds), and (3) Child JA initiation rate (proportion of successfully initiated JA episodes initiated by the child).

	Effects of condition					
Model	Predictors	Estimates	95% CI	SE	*z*/*t* ^a^	*p*
Model 1: JA initiation success rate	(Intercept)	1.12	[0.58, 1.65]	0.27	4.09	<0.001***
	Condition	0.64	[−0.13, 1.42]	0.4	1.63	0.1
Observations	521					
Model 2: mean JA duration	(Intercept)	2.6	[2.43, 2.78]	0.09	29.51	<0.001***
	Condition	−0.01	[−0.26, 0.25]	0.13	−0.07	0.95
Model 3: Child JA initiation rate	(Intercept)	−1.57	[−2.30, −0.84]	0.37	−4.23	<0.001***
	Condition	−0.56	[−1.57, 0.45]	0.51	−1.09	0.28
Observations	377					
Model 1: AIC = 573.23, BIC = 586, *R* ^2^m = 0.02, *R* ^2^c = 0.25, ICC = 0.24, RMSE = 0.4						
Model 2: AIC = 2824.40, BIC = 2840.13, *R* ^2^m = 0.00002, *R* ^2^c = 0.16, ICC = 0.16, RMSE = 0.72						
Model 3: AIC = 348.13, BIC = 359.93, *R* ^2^m = 0.01, *R* ^2^c = 0.36, ICC = 0.35, RMSE = 0.34						

^a^ *t*‐value for linear mixed‐effects model (Model 2), and *z*‐value for generalized linear mixed‐effects models (Models 1 and 3).

*p < 0.05; **p < 0.01; ***p < 0.001.

There was no significant effect of condition for any of the outcome measures: JA initiation success rate (*β* = 0.64, SE = 0.4, *z* = 1.63, *p* = 0.10), mean JA duration (mean JA duration: *β* = −0.01, SE = 0.13, *t* = −0.07, *p* = 0.95), or child JA initiation rate (*β* = −0.56, SE = 0.51, *z* = −1.09, *p* = 0.28). In other words, in JA episodes around both familiar and novel naming events, caregivers were the primary initiators, and the duration and initiation success rate of the JA episodes did not differ by sign familiarity.

We next analyzed whether caregivers timed naming events within JA episodes differently depending on condition (novel/familiar). We predicted that caregivers might be strategic about the type and timing of JA initiations based on the familiarity of the object. Specifically, we predicted that when using familiar signs as labels, caregivers might use the sign itself to initiate JA whereas, when introducing a novel sign, caregivers might first obtain the child's attention, and then label the object. We also predicted that caregivers might repeat novel object labels more often during a JA episode than familiar labels.

Naming events occurred during JA initiation on average 14.71% of the time in the familiar condition and 9.01% of the time in the novel condition. Naming events occurred on average 3.74 s after the onset of a JA episode in the familiar condition, and 4.75 s after the onset of a JA episode in the novel condition. On average, parents named a familiar object 1.65 times per JA episode, and a novel object 2.33 times per JA episode.

**FIGURE 4:**
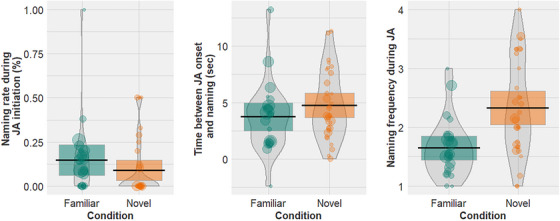
Relationship between Sign Familiarity and (a) Naming rate during JA initiation (proportion of JA episodes in which naming occurred during the initiation step of JA), (b) mean time between JA onset and naming in seconds (the time between the onset of a JA episode and the first naming event in seconds), (c) Naming frequency during JA (the number of repetitions of the object label within each JA episode). Colors represent Condition (green = familiar, orange = novel). Points represent raw data from each dyad, point size represents number of JA episodes contributed per dyad. Bean shows smoothed density curve illustrating the full data distribution. Error bars show frequentist 95% confidence interval.

We analyzed the effect of condition (familiar/novel) on **Naming rate during JA initiation** (proportion of JA episodes in which naming occurred during the initiation step of JA); **Time between JA onset and naming** (the time between the onset of a JA episode and the first naming event in seconds); and **Naming frequency during JA** (the number of repetitions of the object label within each JA episode). In each model the predictor was the condition (familiar/novel), and we included random effects of dyad. For Naming rate during JA initiation, we ran a binomial generalized linear mixed effects model and for Time between JA onset and naming and for Naming frequency during JA we ran linear mixed effects models. The results are illustrated in Figure 4 and Table 4.

**TABLE 4:** Results for (generalized) linear mixed‐effects models: Effect of Condition on (1) naming rate during JA initiation, (2) time between JA onset and naming, and (3) naming frequency during JA.

Effects of condition						
Model	Predictors	Estimates	95% CI	SE	*z/t* ^a^	*p*
Model 1: Naming rate during JA initiation	(Intercept)	−1.87	[−2.33, −1.40]	0.24	−7.81	<0.001***
	Condition	−0.56	[−1.27, 0.15]	0.36	1.54	0.12
Model 2: time between JA onset and naming	(Intercept)	1.69	[1.55, 1.82]	0.07	25.95	<0.001***
	Condition	0.16	[−0.02, 0.35]	0.09	1.78	0.08
Model 3: Naming frequency during JA	(Intercept)	1.65	[1.42, 1.88]	0.11	14.5	<0.001***
	Condition	0.65	[0.31, 0.98]	0.17	3.92	<0.001***
Observations	377					
Model 1: AIC = 271, BIC = 282.79, *R* ^2^m = 0.02, *R* ^2^c = 0.03, ICC = 0.004, RMSE = 0.32						
Model 2: AIC = 680.1, BIC = 695.83, *R* ^2^m = 0.02, *R* ^2^c = 0.17, ICC = 0.16, RMSE = 0.53						
Model 3: AIC = 1188.78, BIC = 1204.51, *R* ^2^m = 0.07, *R* ^2^c = 0.17, ICC = 0.11, RMSE = 1.07						

^a^ *t*‐value for linear mixed‐effects models (Models 2 and 3), and *z*‐value for generalized linear mixed‐effects model (Model 1).

*p < 0.05; **p < 0.01; ***p < 0.001.

Contrary to our predictions, there was no significant effect of sign familiarity/novelty on the Naming rate during JA initiation (*β* = −0.56, SE = 0.36, *z* = −1.54, *p* = 0.12) or on Time between JA onset and naming (*β* = 0.16, SE = 0.09, *t* = 1.78, *p* = 0.08). However, there was a significant main effect on the number of naming events per JA episode (*β* = 0.65, SE = 0.17, *t* = 3.92, *p* < 0.001) indicating that caregivers repeated novel labels more often during JA episodes than familiar labels.

### 3.4 Does Joint Attention Around Naming Events Differ Between First and Subsequent Object Labels?

Our third and final aim was to assess whether the *first* mention of a novel object occurred within JA episodes that had unique properties relative to subsequent JA episodes. We reasoned that an object label is only truly novel the first time it is labelled within a session, and so caregivers might structure JA uniquely when labelling an object—particularly a novel object—for the first time.

To test this, we analyzed the effect of sign familiarity and order of mention on all six JA characteristics we reported above: (1) **JA initiation success rate**, (2) **Mean JA duration**, (3) **Child JA initiation rate**, (4) **Naming rate during JA initiation**, (5) **Time between JA onset and naming**, and (6) **Naming frequency during JA**. We re‐ran all of our models using binomial generalized mixed effects models or linear mixed effects models depending on the nature of the outcome variable (see section 3.3 above), with condition (familiar/novel) and order of mention (first/subsequent) and their interaction as the predictors, and random effects of dyad.

**FIGURE 5:**
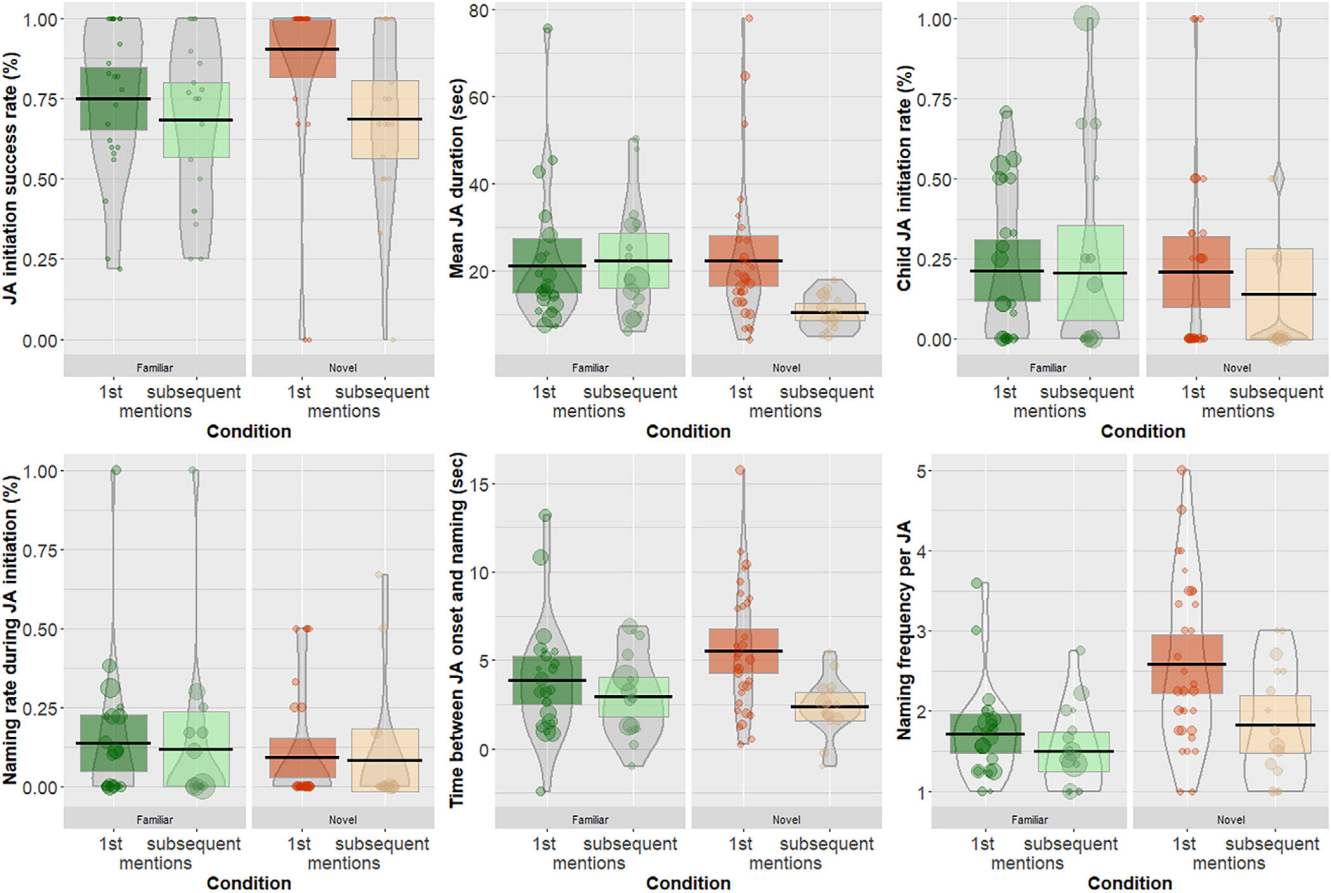
Relationship between condition (familiar/novel), Order of mention (first/subsequent) and (1) JA initiation success rate, (2) Mean JA duration, (3) Child JA initiation rate, (4) Naming rate during JA initiation, (5) Time between JA onset and naming, and (6) Naming frequency during JA. Colors represent Condition (green = familiar, orange = novel) and plots are split by Order of mention (left = first mention, right = subsequent mention). Points represent raw data from each dyad, point size represents number of JA episodes contributed per dyad. Bean shows smoothed density curve illustrating the full data distribution. Error bars show 95% frequentist confidence interval.

The results are illustrated in Figure 5 and Table 5. There was no significant main effect of order of mention (first vs. subsequent object label) in any of the analyses (see Table 5 for all beta values). There was a significant effect of condition (familiar vs. novel) on three of our outcome measures: JA initiation success rate and time between JA onset and naming, neither of which were present in the prior analysis without order of mention (see section 3.3 above), and on the naming frequency during JA, which was also present in the prior analysis. There were no other main effects of condition. More importantly, there was a significant interaction between order of mention and condition for four of our outcome variables: JA success rate, JA duration, time between JA onset and naming, and naming frequency during JA. In all cases, this was because the effect of order of mention was only found in the novel condition (see Figure 5), with significantly more successful JA episodes, significantly longer JA episode durations, significantly more time between JA onset and the naming event, and more frequent object labels per JA episode for the first compared to subsequent mentions of novel labels. No other interactions reached significance.

**TABLE 5:** Results for (generalized) linear mixed‐effects models: Effects of Order of Mention, Condition, and their interaction on (1) JA initiation success rate, (2) Mean JA duration, (3) Child JA initiation rate, (4) Naming rate during JA initiation, (5) Time between JA onset and naming, and (6) Naming frequency during JA.

	Effects of condition and order of mention					
Model	Predictors	Estimates	95% CI	SE	*z*/*t* ^a^	*p*
Model 1: JA initiation success rate	(Intercept)	1.25	[0.69, 1.81]	0.28	4.38	<0.001***
	Order of mention	−0.36	[−0.88, 0.16]	0.26	−1.37	0.17
	Condition	1.41	[0.40, 2.41]	0.51	2.75	0.01**
	Order of mention*Condition	−1.31	[−2.38, −0.24]	0.55	−2.4	0.02*
Observations	521					
Model 2: Mean JA duration	(Intercept)	2.56	[2.38, 2.74]	0.09	28.32	<0.001***
	Order of mention	0.14	[−0.08, 0.36]	0.11	1.26	0.21
	Condition	0.22	[−0.05, 0.48]	0.13	1.65	0.10
	Order of mention*Condition	−0.72	[−1.07, −0.38]	0.17	−4.15	<0.001***
Model 3: Child JA initiation rate	(Intercept)	−1.58	[−2.34, −0.82]	0.39	−4.08	<0.001***
	Order of mention	0.07	[−0.74, 0.88]	0.41	0.18	0.86
	Condition	−0.26	[−1.31, 0.80]	0.54	−0.48	0.63
	Order of mention*Condition	−1.2	[−2.74, 0.34]	.079	−1.53	0.13
Model 4: Naming rate during JA initiation	(Intercept)	−1.81	[−2.32, −1.31]	0.26	−6.99	<0.001***
	Order of mention	−0.22	[−1.10, 0.65]	0.44	−0.5	0.62
	Condition	−0.67	[−1.57, 0.22]	0.46	−1.47	0.14
	Order of mention*Condition	−0.36	[−1.13, 1.85]	0.76	0.47	0.64
Model 5: Time between JA onset and naming	(Intercept)	1.7	[1.56, 1.84]	0.07	25.05	<0.001***
	Order of mention	−0.05	[−0.22, 0.11]	0.08	−0.62	0.54
	Condition	0.27	[0.07, 0.47]	0.1	2.75	0.01**
	Order of mention*Condition	−0.33	[−0.58, −0.07]	0.13	−2.51	0.01*
Model 6: Naming frequency during JA	(Intercept)	0.4	[0.29, 0.51]	0.05	7.53	<0.001***
	Order of mention	−0.09	[−0.23, 0.06]	0.07	−1.18	0.24
	Condition	0.41	[0.25, 0.57]	0.08	5.19	<0.001***
	Order of mention*Condition	−0.34	[−0.56, −0.12]	0.11	−3	0.003**
Observations	377					
Model 1: AIC = 561.42, BIC = 582.69, *R* ^2^m = 0.08, *R* ^2^c = 0.28, ICC = 0.22, RMSE = 0.39						
Model 2: AIC = 2813.03, BIC = 2836.63, *R* ^2^m = 0.05, *R* ^2^c = 0.2, ICC = 0.15, RMSE = 0.71						
Model 3: AIC = 348.93, BIC = 368.59, *R* ^2^m = 0.05, *R* ^2^c = 0.37, ICC = 0.34, RMSE = 0.34						
Model 4: AIC = 274.69, BIC = 294.35, *R* ^2^m = 0.02, *R* ^2^c = 0.03, ICC = 0.01, RMSE = 0.32						
Model 5: AIC = 675.46, BIC = 699.05, *R* ^2^m = 0.05, *R* ^2^c = 0.2, ICC = 0.16, RMSE = 0.52						
Model 6: AIC = 940.84, BIC = 964.44, *R* ^2^m = 0.12, *R* ^2^c = 0.21, ICC = 0.1, RMSE = 0.47						

^a^ *t*‐value for linear mixed‐effects model (Models 2, 5, and 6), and *z*‐value for generalized linear mixed‐effects models (Models 1, 3, and 4).

*p < 0.05; **p < 0.01; ***p < 0.001.

## 4 Discussion

The goal of the present study was to investigate how deaf children learning a sign language achieve joint attention with their caregivers during natural interactions, how these joint attentional episodes scaffold word learning opportunities, and whether characteristics of joint attention predict children's ASL vocabulary size. We also asked whether key aspects of joint attention differ in the context of labeling familiar versus novel objects, and whether caregivers structure joint attention uniquely when labeling an object for the first time within a play session. First, we found that most ASL naming events occur in the context of successful and parent‐initiated joint attention episodes for both familiar and novel objects. The rate at which children and parents successfully responded to JA initiations, as well as how long the dyads maintained their JA episodes, was positively and significantly related to children's ASL vocabulary. Second, while many aspects of joint attention were similar across familiar and novel objects, caregivers repeated novel labels more often than familiar labels during each joint attention episode. Third, when comparing the first versus subsequent object labels within a play session, we found differences in the joint attention success rate, duration of joint attention episodes, time between JA onset and naming, and frequency of object labels—but only when labelling novel objects.

As the starting point for the current study, we speculated that a traditional definition of joint attention as equivalent to joint gaze toward an object may not be sufficient to capture the multimodal nature of joint attention in interaction in sign language. Thus, we applied a JA coding scheme developed specifically to incorporate multimodal behaviors and temporal flexibility in establishing and maintaining joint attention (Gabouer and Bortfeld 2021). As predicted, applying the joint attention scheme to our data revealed that caregivers interacting in ASL effectively establish joint attention with their deaf children when labeling objects in their environment, providing counter‐evidence to claims that interactions with deaf dyads involve lower rates of joint attention (e.g., Prezbindowski et al. 1998). A key feature of the interactions in the current study is that the parent and child shared an accessible language, ASL. Further, the ability of the dyads to successfully achieve joint attention correlated with the children's vocabulary size. Thus, the current findings suggest that to capture joint attention episodes in signing interactions we need to broaden the definition of joint attention to include a range of joint attentional behaviors beyond gaze (e.g., touch), and use a coding scheme that allows for overlap of different behaviors, as long as they are directed at the same object of interest and shared between the two interaction partners.

A comprehensive account of joint attention that allows for the sequential nature of attention to objects and people is particularly important when studying the role of joint attention in language acquisition. In spoken interactions, an object label can be successfully perceived by a child even if the visual attention is not directed toward the caregiver, though multimodal cues certainly support spoken language learning (e.g., Özyürek et al. 2008). However, in signing dyads, while joint gaze does occur, the corresponding object label—which must be perceived visually—must occur in sequence with gaze to an object. As a result, caregivers engaging with their children in sign language accommodate the heightened visual demand by engaging more often in attentional behaviors like waving or touch, and systematically displace their signs to be in the visual space of their child (Holzrichter and Meier 2000). Thus, the canonical definition of joint attention cannot account for interactions amongst signing dyads and, in fact, it is possible that a broader definition of joint attentional behavior might be useful in other contexts too. For example, by broadening our definition of joint attention we might find that joint attentional episodes support vocabulary learning even in cultures that do not prioritize traditional dyadic child‐directed interactions and/or where mutual gaze when in conversation is taboo (e.g., in cultures where speakers seemingly employ “gaze avoidance” at moments where mutual eye contact routinely occurs in other conversational traditions; see Rossano et al. 2009).

We did not find a correlation between our third joint attention characteristic, the children's rate of initiating joint attention, and their vocabulary size. Even though both interaction partners could potentially take either role, joint attention initiator or target of a joint attention initiation, caregivers were the primary initiators of joint attention across dyads. It was surprising to find such high rates of caregiver initiations and low rates of child initiations, as most of the literature reports on a high number of child‐led joint attention episodes (e.g., Kaplan and Yu 2024). We believe this difference is not due to a true divergence in how often joint attention is established around the child's focus of interest. Instead, we speculate that the high rate of parent‐led episodes can be explained by the fact that the scheme applied in this study requires an intentional bid for attention as the act of JA initiation, whereas others also consider looks or touches to the object, which may not themselves be intentional of sharing attention with the interaction partner. It is possible that many of these caregiver‐initiated joint attention episodes are indeed child‐led (e.g., child looks at an object) but that the first intentional behavior is by the caregiver. Further work is needed to consider the impact of different types of coding schemes on the description of initiation behavior in joint attention.

The caregivers in our dataset repeated labels more often when interacting with novel objects, suggesting that caregivers may have been intentionally providing multiple instances of labels for novel objects within JA episodes to support word learning. None of our other JA measures was affected by sign familiarity per se, but more effects emerged when we also considered first compared to subsequent labeling instances within novel and familiar naming events. Within the sessions around novel objects, there were significantly more successful JA episodes, significantly longer JA episode durations, a significantly longer time between onset and naming, and significantly more repetitions of naming events for the first compared to subsequent mentions of novel labels. None of these effects were present in the familiar sessions. These results suggest that the parents were highly attuned not only to the visual attention of their child at the moment of object labeling, but also to the (assumed) novelty of a sign, and in particular to the amount and timing of attention needed to ensure that the child perceived a novel object label the first time it was provided (for similar results for dyads interacting in spoken language, see Chen et al. 2021). In other words, the parents—and perhaps also children—were modifying their behavior in joint attentional episodes in such a way that maximized the opportunities provided by the joint attention context to learn the meaning of novel signs.

The current study did have limitations. In particular, we did not assess the same participant's behavior in familiar and novel toy play sessions, so we were not able to directly compare how individual caregivers modify their behavior based on the familiarity of the object being labelled. Further, we did not explicitly assess whether the familiar object labels were indeed known by the individual children but rather assumed their familiarity by their prevalence, which may have led us to underestimate the effects of novelty. More importantly, the study provides only a snapshot of US‐American caregiver‐child interaction around objects in one language (ASL) in a limited context (toy play with specified objects). The participants were predominantly White and had a high level of education, limiting our ability to generalize to a more diverse sample. Nevertheless, our findings from dyads with deaf children interacting in ASL illustrate that research on joint attention and language acquisition can profit from broadening the definition of joint attention and considering the multimodal nature of dyadic interaction. The present study lays the groundwork for future research exploring the behaviors involved in initiating and maintaining joint attention episodes in a more fine‐grained manner. Exploring how visual, auditory, and tactile behaviors interact could offer important insights into how different JA strategies lead to successful JA episodes (Depowski et al. 2015) and could support our further understanding of modality specific patterns of caregiver and child behaviors.

In conclusion, the analyses in the present study give us a better understanding of how caregivers shape the interactional environment in which children encounter labels to the objects in their surroundings. Investigating how the properties of joint attention episodes vary under different conditions and across modalities offers insight into broader questions about how both caregivers and children may adapt their behaviors in dyadic interaction based on the sensory profiles of the interaction partners. The findings from our study add to the growing body of evidence that caregivers and children interacting in ASL engage in frequent and successful episodes of joint attention, and this engagement in joint attention contributes to children's word learning in ASL. Dyads’ engagement in joint attention differed depending on the assumed familiarity of the sign and by the order of mention of the object label, which suggests that caregivers actively and dynamically modulate joint attention to support children's novel sign learning. Our study contributes to our understanding of the importance of joint attention, broadly and appropriately defined, as a special type of interaction that supports early language learning across cultures, languages, and modalities.

## Ethics Statement

All study procedures were approved by the Institutional Review Board at Boston University.

## Conflicts of Interest

The authors declare no conflicts of interest.

## Data Availability

The data that support the findings of this study are openly available in OSF at https://osf.io/wmeck/, reference number DOI 10.17605/OSF.IO/WMECK.

## Endnotes

1 We ran three separate linear regressions instead of one multiple regression that included all three predictor variables because we were interested in whether each JA characteristic is a significant predictor regardless of whether it shares variance with the other predictors. Note, that the results from one multiple regression model showed that JA initiation success rate was a significant predictor, but mean JA duration of successful JA episodes and Child JA initiation rate were not. Further, note that we have not accounted for age in these models, as child's age is highly correlated with child vocabulary (correlation = 0.75). If we include age as a predictor in either one multiple regression or the three separate regressions JA initiation success rate remains a significant predictor of child production vocabulary.
